# Comparative efficacy of remotely delivered mindfulness-based eating awareness training versus behavioral-weight loss counseling during COVID-19

**DOI:** 10.3389/fpsyg.2023.1101120

**Published:** 2023-05-17

**Authors:** Carla Ugarte Pérez, Claudia Cruzat-Mandich, Álvaro Quiñones Bergeret, Dafne Díaz-Tendero, Marcela Gallegos, Aurora A. Gil, Antonio Cepeda-Benito

**Affiliations:** ^1^Centro de Estudios de la Conducta Alimentaria (CECA), Escuela de Psicología, Universidad Adolfo Ibáñez, Peñalolén, Chile; ^2^Department of Social Science, University of Tarapacá, Iquique, Chile; ^3^Departamento de Nutrición y Bariátrica, Clinica Las Condes, Santiago, Chile; ^4^Eating Disorders Unit, Psychiatry Department, Faculty of Medicine, Pontificia Universidad Católica, Santiago, Chile; ^5^Department of Psychological Science, University of Vermont, Burlington, VT, United States; ^6^Departamento de Psicología, Personalidad y Tratamiento, Universidad de Jaén, Jaén, Spain

**Keywords:** binge eating, emotional eating, external eating, mindful eating, mindfulness-based eating awareness training, randomized controlled trial, COVID-19, telehealth

## Abstract

**Introduction:**

Dysregulated eating (emotional eating, cue-elicited eating, and dietary restraint and restriction) has been linked to being overweight or obese. The present investigation used a random controlled trial (RCT) to test the differential efficacy of remotely delivered Mindfulness-Based Eating Awareness Training (MB-EAT) and Behavioral Weight Loss (BWL) counseling.

**Methods:**

The sample was recruited through advertisements that offered help to people “with problems controlling their eating” or “interested in improving their relationship with food” (n = 135).

**Results:**

Retention was low in both groups (42%), but not dissimilar to retention rates reported in related clinical trials delivered “in person.” Among the participants who completed treatment, we found no between-group differences in any of the treatment outcomes, but participants in both groups experienced significant increases in eating-related mindfulness [Mindful Eating Questionnaire (MEQ) and awareness [Multidimensional Assessment of Interoceptive Awareness (MAIA), and significant decreases in unhealthy eating patterns [Dutch Eating Behavior Questionnaire (DEBQ); Binge Eating Scale (BES), and weight over the course of treatment. Participants in both groups also experienced increases in self-reported depression and anxiety symptoms [Hospital Anxiety and Depression Scale (HADS)], although these increases likely reflected normative changes observed in the population at large during COVID-19.

**Discussion:**

Overall, the results suggest that dysregulated eating and weight loss intervention delivered remotely via teleconference can be effective.

## 1. Introduction

Excessive body weight is a well-known risk factor for a myriad of chronic diseases, including cardiovascular disease, diabetes mellitus, chronic kidney disease, many cancers, and musculoskeletal disorders (GBD, 2015). It has been estimated that being overweight or obese may contribute globally to over 7% of all deaths, and to nearly 5% of all disability-adjusted life-years, with nearly 40% of these deaths and disability years related to just being overweight (GBD, 2015). Being overweight or obese also carries considerable psychological burdens, with people with overweight having an increased risk for anxiety and substance-use disorders, and people with obesity having an increased risk for anxiety, mood, alcohol use, and personality disorders (Petry et al., 2008). In addition, people with overweight or obesity are subjected to pervasive public and societal stigmatization and discrimination, which also have grave consequences for their well-being and quality of life (Puhl and Heuer, 2010). For example, there is strong evidence suggesting that internalized weight stigma is a significant risk factor for depression, anxiety, substance abuse, and suicidality (Rubino et al., 2020).

### 1.1. Dysregulated eating, overweight, and obesity

Among many other factors, researchers have long described three different types of eating or eating patterns that impact overeating and weight gain (see van Strien et al., 1986). Originally described as “psychosomatic” eating (e.g., Kaplan and Kaplan, 1957), “emotional eating” refers to eating driven by emotional states, such as anger, anxiety, and stress; all of which mimic to some extent hunger’s physiological interoceptive signals. Schachter et al. (1968) described “external eating” as occurring in response to food-related stimuli independently of the individual’s internal state of hunger or satiety. Finally, dietary restraint and restriction refer to individuals’ efforts to limit their caloric intake and control their weight (e.g., Polivy and Herman, 1985). It has been theorized that when self-regulation of intense dieting breaks down, individuals become disinhibited, and binge eat to satiate their accumulated hunger and/or cope with their emotional dysregulation (Polivy and Herman, 1985). It is also likely, that excessive and prolonged restrictive dieting may lead to poorer recognition of interoceptive hunger signals, and thus increase consumption through increased emotional and external eating (Polivy and Herman, 1976).

Although the theorizing cited above may appear dated, these original theories continue to be cited in current research, and they have stood the test of time and empirical testing. For instance, Elliston et al. (2017) monitored 51 adults with overweight and obesity over 2 weeks using ecological momentary assessment (EMA). These authors found that internal (negative affect) and external (food proximity, specific social contexts) cues increased the probability of eating and snaking. In a review of the literature, van Strien (2018) found that high dietary restraint, poor interoceptive awareness, alexithymia, and emotion dysregulation were all likely causal factors of emotional eating. This author concluded that the treatment of people with overweight or obesity should focus on emotion regulation skills rather than calorie-restricted diets. Willem et al. (2019) compared people with severe obesity, moderate obesity, and normal weight and found people with severe and moderate obesity self-reported less interoceptive awareness and more emotion dysregulation than participants with normal weight.

There are various phenomena congruent with the hypothesis that being overweight or obese is linked to the three “kinds” of eating described above. For example, although individuals of any weight or body mass index (BMI; kg/m^2^) may engage in emotional, external, or restrictive eating, their occurrence is common and frequent among those who are overweight (BMI > 24.9) or obese (BMI > 29.9; Devonport et al., 2019; Opichka et al., 2019). Not surprisingly, emotional, external, and/or restrictive eating correlate with the consumption of highly energy-dense foods, with weight gain over time (Hummel et al., 2018; Halali et al., 2020), and with binge-eating disorder (BED) symptoms (Kristeller et al., 2014). In addition, BED, which is characterized by eating unusually large amounts of food in a relatively short period of time and feeling out of control while eating, includes symptoms such as eating to handle emotional distress, frequent dieting, strong reactivity to food cues, and dysregulation of interoceptive awareness related to appetite and satiety (e.g., see also Sobik et al., 2005; McIntosh et al., 2006).

### 1.2. Treating mindless and dysregulated eating

Although behavioral and pharmacological, weight-loss treatments can be effective at end-of-treatment (EOT) and short-term follow-up, maintaining weight loss gains long term is rare (Adams, 2012; Wirth et al., 2014; Montesi et al., 2016; Halali et al., 2020). Even bariatric surgery, which quickly achieves substantive weight-loss outcomes, is also associated with long-term failure to keep BMI at EOT levels (Courcoulas et al., 2013, 2014, 2018). In an attempt to improve the effectiveness of weight loss interventions, clinicians and researchers have incorporated mindfulness training to treat dysregulated eating, including BED (e.g., Rott et al., 2008; Kristeller et al., 2014; Miller et al., 2014; Kristeller, 2015). In theory, mindfulness-based interventions could address the dysregulated, mindless eating that leads to excessive eating and weight gain (e.g., Kristeller and Hallett, 1999; Kristeller, 2015) and replicate the success of mindfulness training in treating other dysregulation-related disorders such as anxiety, depression (see Hofmann et al., 2010) or substance abuse (e.g., Bowen et al., 2009).

Kristeller and Hallett (1999) were the first to test the feasibility and potential efficacy of a novel BED intervention they coined Mindfulness-Based Eating Awareness Training (MB-EAT). Since then, various investigations have reported that MB-EAT can improve the regulation of eating-related behaviors (Kristeller and Wolever, 2011; O'Reilly et al., 2014; Schnepper et al., 2019; Carrière et al., 2022) and weight loss effects (Olson and Emery, 2015; Chung et al., 2016; Fuentes Artiles et al., 2019; Schnepper et al., 2019), even if with occasionally mixed results (Daubenmier et al., 2016; Tapper, 2017).

Mindfulness can be described as the intentional act of paying conscious, continuous attention to internal experiences with an open, non-judgmental attitude (Kabat-Zinn, 1990, 2015). Being mindful involves regulating and directing one’s attention to the “here-and-now” and requires “acceptance” without judging nor attempting to control that which is perceived, felt, sensed, or thought (Bishop et al., 2004; Jenkins and Tapper, 2014; Kiken et al., 2015; Tapper, 2017, 2018). It has been proposed that through practice, mindfulness leads to “decentering,” or the ability to notice and observe negative thoughts and feelings without persevering nor obsessing on what they may signify, consequently diminishing the distress that usually accompanies the negative thoughts and feelings that damage mental health (Bennett et al., 2021). Therefore, MB-EAT encourages directing nonjudgmental attention to the internal sensations, feelings, and thoughts that are present or emerge while eating (Kristeller and Wolever, 2011; Mantzios and Wilson, 2014). For example, MB-EAT coaches participants to notice and objectively accept the sensory qualities of foods, including their smell, texture, and flavor, as well as the interoceptive signals associated with cravings, hunger, and satiation (Hendrikse et al., 2015; Baer et al., 2019). Thus, MB-EAT directly addresses the psychophysiological mechanisms implicated in dysregulated, excessive eating and, consequently, weight gain (e.g., Warren et al., 2017; Dutt et al., 2019; Czepczor-Bernat et al., 2020; Mantzios et al., 2020; Carrière et al., 2022).

### 1.3. COVID-19 confinement: impact on weight gain, disordered eating, obesity, and health care delivery

The COVID-19 pandemic was associated with increases in BMI globally (NIH US National Library of Medicine, 2020; Zachary et al., 2020; Bhutani et al., 2021), including Chile (IPSOS, 2021). This is hardly surprising given that COVID-19 restrictions worldwide profoundly disrupted people’s lives (e.g., Hernández-López et al., 2021), including their eating (Abbas and Kamel, 2020; Touyz et al., 2020) and movement and exercising habits (Rodríguez et al., 2020). Increases in BMI during the COVID-19 pandemic have been linked to increases in sedentarism (Rodríguez et al., 2020), the consumption of ultra-processed foods (Di Renzo et al., 2020; Phillipou et al., 2020; Sidor and Rzymski, 2020; Ammar et al., 2021), and increases in the rate and prevalence of stress-induced binge eating (Phillipou et al., 2020; Freizinger et al., 2022), particularly among people with overweight and obesity (Almandoz et al., 2020).

Confinement and social isolation bring about stress and increase the risk for disordered eating (Nevanperä et al., 2012; Hou et al., 2013; Järvelä-Reijonen et al., 2016), which is often used as a mechanism to cope with negative affect (Chen et al., 2020; NIH US National Library of Medicine, 2020; Touyz et al., 2020; Robinson et al., 2021). Thus, many studies anticipated and found that COVID-19 confinements and social restrictions would bring about overeating, binge eating (Cecchetto et al., 2021), and progressive weight gain (Di Renzo et al., 2020; Phillipou et al., 2020; Ammar et al., 2021; Pellegrini et al., 2020).

The COVID-19 pandemic also brought about a transformation in mental and behavioral health care (Mann et al., 2020; Pierce et al., 2021). For example, Pierce et al. (2021) documented that the delivery of psychological services in the U.S. increased 12-fold during the COVID-19 pandemic. These authors estimated that during the pandemic up to three-thirds of U.S. licensed psychologists conducted 100% of their clinical work remotely, and up to one-third of this work will remain remote post-COVID-19 (Pierce et al., 2021).

### 1.4. Eating disorders, overweight, and obesity in Latin America, including Chile

In a systematic review and epidemiological meta-analysis, Kolar et al. (2016) found point-prevalence rates of 1.16% for Bulimia Nervosa, and 3.53% for binge-eating disorder (BED) across six Latin American countries: Argentina, Brazil, Chile, Colombia, Mexico, and Venezuela. Kolar et al. (2016) also noted that the point-prevalence, BED rates they found were higher than those usually reported for Western countries. In addition, Kolar et al. (2016) observed that their findings were unsurprising given that BED is highly prevalent among people with overweight or obesity, particularly among females, and prevalence rates of overweight and obesity are higher in Latin America than in Western countries. Gaete and López (2020) observed that lifetime, risk-prevalence studies estimate that between 7.4 and 12% of all Chilean adolescents (8.3 to 23% for girls) will develop an eating disorder.

Among Latin American countries, Chile is the richest or most economically developed country (as defined by Gross Domestic Product in US dollars *per capita*; OECD, 2022), but Chile also has one of the highest, overnutrition prevalence rates in Latin America, with 76% of adults and 58% of children being overweight or obese (OECD Obesity Update, 2017; JUNAEB, 2022; Ministerio de Salud Chile, 2022). Chile’s favorable economic growth may in part explain why Chile’s overnutrition trends are so high. Cultural modernization brought about by economic growth contributes to lifestyles and behavioral changes that include a high degree of sedentarism and overconsumption of highly energy-dense foods (Ministerio de Salud, 2017). Modern ubiquity and consumption of ultra-processed foods in developed countries instrumentally contribute to marked increases in overweight and obesity rates [Ministerio de Salud, 2017; NCD Risk Factor Collaboration (NCD-RisC), 2017; OECD Obesity Update, 2017; Fox et al., 2019; Goh et al., 2020; World Health Organization, 2022]. Ultra-processed foods are industrially manipulated foods made to be highly palatable through the combination of synthesized and refined ingredients that artificially makes them very high in caloric content (Monteiro et al., 2019).

### 1.5. Rationale and aims of the current study

The present investigation emerged out of the need to cope with COVID-19 impacts on eating and weight gain, as well as to cope with the difficulty of continuing to offer mental and behavioral health services related to overeating and weight gain in people with overweight and obesity. Thus, we felt compelled to adapt to remote delivery an intervention we routinely offer and implement (MB-EAT), and use this challenge as an opportunity to examine its efficacy versus a behavioral-based treatment (BWL). To accomplish our goals, we adapted the MB-EAT and BWL interventions to remote delivery via teleconference, randomly assign participants to the two treatments, and compared baseline to end-of-treatment (EOT) changes in various outcome variables (including BMI, overall and eating-related awareness, and disordered eating).

Past investigations evaluating the effects of MB-EAT on eating regulation and weight loss have varied considerably. Some studies have not used randomized control trial (RCT) designs (e.g., Chung et al., 2016; Mason et al., 2018; Wnuk et al., 2018; Jafari, 2020), while others have used RCT designs with just waitlist controls (e.g., Timmerman and Brown, 2012; Stites et al., 2015; Kristeller and Jordan, 2018), and four RCT studies used various kinds of active controls (Miller et al., 2012; Kristeller et al., 2014; Mason et al., 2018; Salvo et al., 2022). Generally, studies using waitlist controls have found MB-EAT to produce efficacious outcomes regarding weight loss at EOT assessments, whereas those comparing MB-EAT with active control interventions have not found differential efficacy but reported comparable and significant pre-to-post treatment effects across interventions. We chose an active control intervention (BWL) because wait-list controls tend to overestimate effect sizes (e.g., Cunningham et al., 2013; Laws et al., 2022), and active-treatment controls are generally considered to provide more rigorous efficacy and effectiveness tests than wait-list controls (e.g., Steinert et al., 2017).

## 2. Methods

### 2.1. Procedures

#### 2.1.1. Recruitment

We posted social media announcements on Facebook, Instagram, and LinkedIn profiles connected to the authors’ professional and institutional networks. The posts sought individuals with BMIs >24 and “with problems controlling their eating” or “interested in improving their relationship with food.” The wording for the advertisements was suggested by experienced counselors who work with people with overweight and obesity who seek help with the goal or desire to lose weight. We felt those expressions would resonate with a broad range of people with high levels of dysregulated eating. The advertisement also provided potential participants with instructions to calculate their BMIs. Participants contacted us via email, and we called them back to set up an eligibility screening via Zoom. To participate, respondents had to be at least 18 years old, have a BMI >24.9, and report a lack of control over their eating (being concerned about their binge eating, external eating, emotional eating, and/or overeating while satiated). Participants were excluded if they were pregnant or had given birth within 6 months or were breastfeeding. Participants who had had bariatric surgery in the previous 12 months were also excluded. To increase the homogeneity of the sample, individuals with a likely diagnosis of bulimia nervosa were excluded from the study.

#### 2.1.2. Treatment enrollment and delivery

Eligible participants received an email that included the informed consent form and a code-protected link with access to the outcome baseline measures. The baseline survey required participants to report their age, gender, weight (kg), and height (m), and complete all the treatment outcome variables. Those who completed the baseline survey were randomly assigned to the MB-EAT or Psych-Ed interventions, which were run concurrently on Tuesdays and Thursdays in weekly, 2-h sessions over an 8-week period. Both interventions were carried out through the Zoom. There were three enrollment waves, with a similar number of participants assigned to the two interventions per wave (*n* = 14 to 18 per group). The three cycles took place in successive order between March 1 and August 12, 2021. All but the second half of the last cycle coincided with the most pronounced COVID-19 infection spikes observed in Chile to date (Aguilera et al., 2022). Both interventions were led by the same team of specialized licensed professionals, which consisted of a psychiatrist, a psychologist, and a nutritionist, all with extensive training and experience in eating disorders, weight loss treatments, and in this type of intervention (Díaz-Tendero et al., 2018, 2019; Gallegos et al., 2019).

### 2.2. Participants

**Figure 1:**
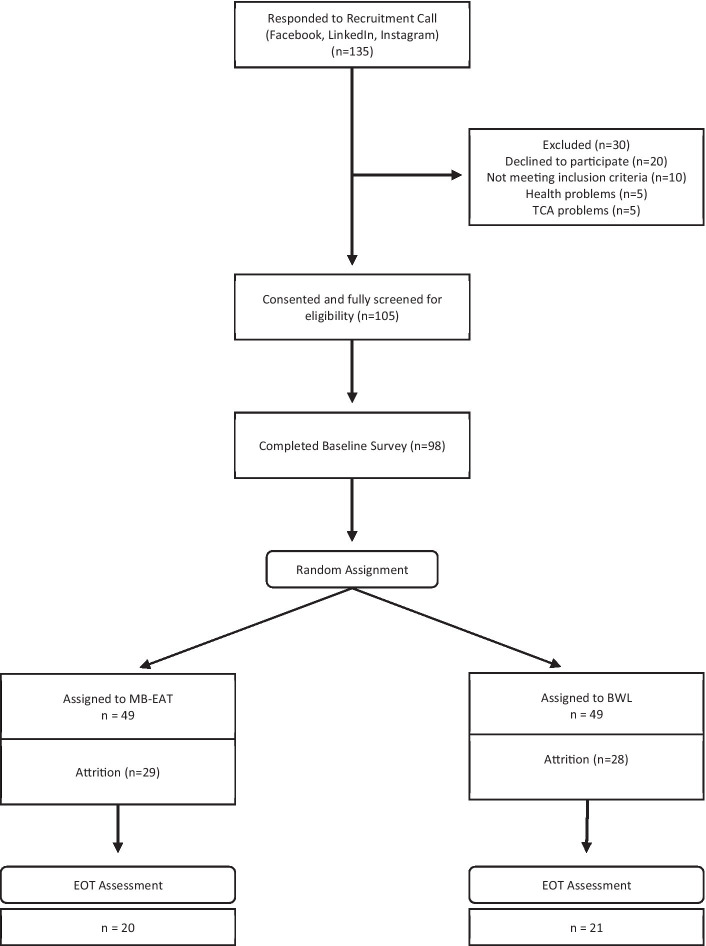
Participant flow chart.

Figure 1 depicts recruitment and attrition flows. Of the participants who responded to our social media advertisements (*n* = 135), some declined or did not respond to participate in the screening interview (*n* = 20), and others were ineligible because they reported bulimic-specific symptoms (*n* = 5), or other severe health problems (*n* = 5). Ineligible participants were referred to appropriate services.

Of the 105 screened and eligible participants, 98 (93%) completed the BT survey. The gender distribution of this baseline sample included 84 women (76%) and 14 men (24%), reported an average age of almost 35 (*M* = 34.8, *SD* = 10.2), and had an average BMI near 32 (*M* = 31.71, *SD* = 5.06). Attrition over the 8-week-long intervention was high, and only 41 (42%) participants remained and completed end-of-treatment (EOT) outcome measures. The gender distribution in the retained group (female n = 36, male *n* = 5) was not significantly different from the attrition group (female n = 48, male *n* = 9; *Chi^2^* [1] = 0.252; *p* = 0.612); and average BMI was nearly identical in both groups (*Mdiff* = 0.62, *SE* = 1.04; *t* [96] = 0.60, *p* = 0.553). However, those lost to attrition were significantly younger (*M* = 32.7, *SD* = 9.0) than those retained (*M* = 37.8, *SD* = 11.1; *t* [96] = 2.51, *p* = 0.014). There were no differential attrition patterns between the two treatments, but most participants lost to follow up stopped attending after 4 sessions (55 out 57), with most of these attending 2 or fewer sessions (30/55). Two participants, one per group, attended all the sessions but did not complete the EOT outcome measures.

### 2.3. Interventions

#### 2.3.1. Mindfulness-based eating awareness training

The MB-EAT intervention was modeled after Kristeller and Hallett (1999), Kristeller and Wolever (2011), and Kristeller et al. (2014) and is described in detail in Table 1. The purpose of the intervention was to increase mindful awareness of eating-related experiences and to reduce emotionally and contextually triggered eating. Activities were practiced in session and prescribed to be practiced at home for the duration of the intervention. The activities focused on increasing awareness of (a) physical hunger and physical satiety cues, (b) the amount of food consumed, and (c) the physical, cognitive, social–environmental, and emotional triggers of binging. Meditation exercises included (a) general (breath/open awareness) mindfulness, (b) guided eating meditations, and (c) brief, in-the-moment meditation strategies to practice at mealtime and throughout the day.

**Table 1:** Organization MB-EAT sessions.

Objectives and learning goals	Mindfulness exercises and activities	
	General	Eating specific
1. Introduction to Mindfulness: Full awareness and internalization. Internal and external wisdom. How the mind works.	Formal meditation.	Raisin exercise.
2. Introduction to Mindful Eating: Becoming aware of eating: how, when and what.	mini Meditation.	Calories dense foods.
3. Autoregulation and emotions: Food and emotions exploration	Wisdom meditation	Hunger awareness: physical vs. emotional
4. Body signals I: Fostering self-compassion vs. self-criticism	Healing self-touch.	Taste satisfaction and satiety
5. Body signals II: Increase awareness of why we eat: “Eating like a newborn”	Body scan	Fullness Awareness
6. Conscious choices: Increase awareness of how and what we eat	Mini meditation	Buffet meal.
7. Body and physical activity: Conscious and kind relations with the body	Everyday awareness/Chair yoga and stretching	
8. Closure: recapitulation	Closure meditation	Meditation and eating triggers

#### 2.3.2. Behavioral weight loss counseling

The BWL control intervention rested on a cognitive-behavioral (mechanistic) explanation of the binge-eating cycle and taught cognitive-behavioral coping strategies to develop healthy habits and cope with distress and daily challenges (see Table 2). An important goal of the intervention was to familiarize participants with cognitive behavioral theory and gain a barebones understanding of how thoughts feelings and behaviors are connected, as well as how habits develop (particularly around eating and food). As with MB-EAT, participants learned and practice the activities they would then need to carry out as homework assignments for the duration of the intervention.

**Table 2:** Organization of BWL intervention.

Objectives and learning goals	Activities	
	In session	Homework
1. Introduction: Definition of “weight” and factors that impact body weight.	How to create and use a weight chart	Track weight and diet
2. Understand the diet-binge cycle. Caloric intake effects on weight gain.	Read and interpret food labels	Challenge: Reduce portions. Read food labels at home
3. Hunger: types and other eating triggers.	Small group discussion: Eating without hunger. Progressive relaxation.	Practice progressive relaxation
4. Emotional eating. Preventing binge eating. Physical signs of satiety.	Behavioral chain analysis	Write own behavioral chain description regarding overeating
5. Hunger and satiety. Mechanisms of eating regulation. Feeling “full.”	Review and analysis of personal weight charts	Apply a change to an eating habit or custom (e.g., change foods).
6. Buffets: Contextual impact and tools to cope with free access to food abundance	Review buffet exercise	Do not eat while exposed to an overabundance of palatable foods.
7. Importance of physical activity. Physical activity and weight control	Counting steps	Estimate average daily steps per week and commit to increase by 10%
8. Closure: Review of learning objectives and activities	Analysis of achievements over barriers.	Commit to maintain gains and identify new challenges

### 2.4. Measures

#### 2.4.1. Mindful eating questionnaire

The MEQ is a 28-item, five-factor measure that was developed to assess, “A non-judgmental awareness of physical and emotional sensations while eating or in a food-related environment” (p. 1440; Framson et al., 2009). The five factor-derived scales, respectively, measure Disinhibition, Awareness, External Cues, Emotional Response, and Distraction. Respondents indicate on a 4-point Likert-type scale how rarely or frequently the item applies to them, from “Never/Rarely” (1) to “Usually/Always” (4), which can produce total scores ranging from 28 to 112. There is ample evidence of sound internal consistency reliability and construct validity for the Spanish version of the MEQ with several adult community samples from Chile (e.g., González, 2018; Carvajal and Sánchez, 2021), as well as Spanish-speaking adult and adolescent samples in the US (Goodwin et al., 2017) and children samples from Chile (Gayoso et al., 2021). In our sample, the internal consistency of the Spanish version of the MEQ was also excellent (Cronbach α = 0.80 at both pre and post-EOT).

#### 2.4.2. Multidimensional assessment of interoceptive awareness

The MAIA is a 32-item, eight-factor, self-report measure that assesses interoceptive body awareness (Mehling et al., 2012). Each of the eight factors of dimensions of the MAIA is comprised three to seven items that assess interoceptive awareness characterized as, or relating to, Noticing, Non-Distracting, Non-Worrying, Attention Regulation, Emotional Awareness, Self-regulation, Body-Listening, and Trusting. Items are scored using a six-point, Likert-type scale from “Never” (0) to “Always” (5), which produces total scores that can range from 0 to 160. There is a Spanish version of the scale that was validated in Chile with an adult, community sample and yielded excellent internal consistency (Cronbach α = 0.90) and construct validity (Valenzuela-Moguillansky and Reyes-Reyes, 2015). In our hands, the internal consistency of MAIA total scores was also excellent (Cronbach α = 0.92 and 0.93 at pre- and post-treatment).

#### 2.4.3. Dutch eating behavior questionnaire

The DEBQ is a 33-item measure that assesses three types of eating-related behaviors, restrained, emotional, and external eating (van Strien et al., 1986). Items are scored using a five-point, Likert type scale from “Never” (1) to “Very Frequently” (5). Summed, item scores produce total scores that can range from 33 to 165. The DEBQ has been translated to Spanish and was tested with a Chilean adult, community sample (Andrés et al., 2017). Andrés et al. (2017) reported excellent internal consistency for the total scores (Cronbach α = 0.87 to 97), as well as ample construct validity. In our sample, total scores were also highly reliable (Cronbach α = 0.90 and 0.92 at pre- and post-treatment).

#### 2.4.4. Binge eating scale

The BES is a 16-item questionnaire that assesses key behavioral (e.g., eating large amounts of food) and affective/cognitive symptoms (e.g., guilt) that precede or follow binge episodes (Gormally et al., 1982). Each item contains 3 to 4 multiple-choice statements that indicate different severity levels for each measured symptom. Participants select the statement that best describes their experience. Total scores can range from 0 to 46. A Spanish version of the BES has been validated in adult populations with Mexican (Zúñiga and Robles, 2006) and Spanish samples (Escrivá-Martínez et al., 2019), yielding excellent internal consistency (Cronbach α = 0.87 to 0.92) and adequate to excellent construct validity in both samples. In our Chilean sample, total scores were also highly reliable (Cronbach α = 0.91 and 0.86 at pre- and post-treatment).

#### 2.4.5. Hospital anxiety and depression scale

The HADS is a 14-item instrument that assesses anxiety and depression symptoms using a 4-choice format that asks participants to select frequency or intensity statements that describe their experience with different symptoms (Zigmond and Snaith, 1983). Each item is scored in scale that ranges from 0 (e.g., “Never” or “Not at all”) to 3 (e.g., “Very often” or “Definitely). Summed items produce a total score that can range from 0 to 42. The HADS has been translated to Spanish and validated with many different Spanish-speaking community samples from Spain (Terol-Cantero et al., 2015) and at least one from Chile (Villoria and Lara, 2018). The Spanish HADS has consistently shown its scores fit a two-factor structure that, respectively, assess anxiety and depression, as well as evidence of adequate to strong internal consistency (Cronbach a = 0.80 to 0.87) and construct validity (see Terol-Cantero et al., 2015). In our sample, total scores were also reliable (Cronbach α = 0.87 and 0.84 at pre- and post-treatment).

## 3. Results

### 3.1. Attrition

As indicated above, attrition was very high, with only 42% of enrolled participants remaining in treatment and completing the EOT measures. Table 3 presents the means and standard deviations for both attrition and retained participants within each treatment condition. Two-way, univariate analyses of variance (ANOVAs) compared mean scores for age, BMI, and the six EOT variables, with treatment (MB-EAT vs. BWL) and attrition (retained vs. lost) as the two between-subjects factors. These analyses did not reveal significant effects for the treatment by attrition interaction (all *F*s[3, 94] < 2.45; *p* > 0.120), or for the treatment factor (all *F*s[1, 94] < 2.45; *p* > 0.120). However, there were significant attrition effects for age (*F*[1, 94] = 6.26; *p* = 0.014; *h^2^ = 0.*062), HADS scores (*F*[1, 94] = 5.18; *p* = 0.025; *h^2^* = 0.053), and BES scores (*F*[1, 94] = 11.54; *p* = 0.001; *h^2^* = 0.110), with participants in the attrition group being significantly younger, and reporting greater symptoms of anxiety/depression and binge eating than the retained group (see Table 3).

**Table 3:** Means (standard deviations) of outcome variables at baseline for retained and attrition participants in the mindfulness-based eating awareness training (MB-EAT) and behavioral weight loss (BWL) groups.

		Retained (*n* = 41)	Attrition (*n* = 57)
Outcome Variable	Intervention	M (SD)	M (SD)
Body mass index	MB-EAT	33.37 (6.19)	31.70 (3.88)
	BWL	30.71 (5.41)	31.22 (4.62)
Mindful eating questionnaire	MB-EAT	69.00 (11.22)	66.14 (9.26)
	BWL	70.37 (13.73)	69.52 (11.39)
Multidimensional assessment of interoceptive awareness	MB-EAT	88.31 (18.59)	81.07 (29.17)
	BWL	82.86 (24.84)	84.24 (24.12)
Dutch eating behavior questionnaire	MB-EAT	111.43 (17.56)	119.92 (17.50)
	BWL	111.75 (20.13)	114.10 (19.93)
Binge eating scale	MB-EAT	20.00 (7.30)	26.29 (8.84)
	WBL	19.74 (10.52)	26.62 (10.41)
Hospital anxiety and depression scale	MB-EAT	12.14 (5.45)	16.75 (6.97)
	BWL	15.42 (7.31)	17.24 (7.28)

MB-EAT-Retained (*n* = 21); BWL-Retained (*n* = 20); MB-EAT-Attrition (*n* = 28); BWL-Attrition (*n* = 29).

To further investigate what individual characteristics could have increased the probability of attrition at EOT, we conducted a binary logistic regression to predict group membership (attrition = 1; retention = 0). Group membership was regressed on age, treatment group, BMI, and the five continuous outcome variables. The model fit was statistically significant (*Chi^2^* [8] = 18.61; *p* = 0.017) and classified correctly 71% of the participants. The model showed worse specificity than sensitivity as it classified correctly 62.5% of those retained but classified correctly 77.2% of those lost to attrition. Of all the predictors in the model, only the regression slope associated with BES scores, or binge-eating-related symptoms (*β* = 0.116, *p* = 0.012), significantly and positively increased the odds ratio (*OR*) of participants belonging to the attrition group (*OR* = 1.12, *95%CI* = 1.03 to 1.23). Notably, neither age nor HADS scores were predictive of attrition in the model with all the predictors entered simultaneously, perhaps because of shared covariance as younger participants tended to report higher binge-related symptoms (*r* = −0.23, *p* = 0.021), and HADS and BES scores were strongly and positively correlated (*r* = 0.57, *p* < 0.001).

### 3.2. EOT outcomes

We conducted a repeated-measures analysis of variance with BMI and each of the five additional EOT outcome measurements. The baseline and EOT assessments constituted the within-subjects factor. Intervention type (MB-EAT vs. BWL) was the between-subjects factor. Without exception, each of the repeated-measures ANOVAs yielded a statistically significant effect for the within factor, but no significant effect for the between factor nor the within by between factors interaction.

Table 4 includes the means and standard deviations for each treatment group at baseline and EOT; and Table 5 summarizes the ANOVA results. Except for symptoms of anxiety and depression, baseline to EOT changes happened in the desirable direction. That is, participants reported greater mindfulness of their eating restraint, and of their emotional and cue-elicited eating (MEQ scores), as well as greater interoceptive awareness (MAIA scores) at EOT than at baseline. The effect sizes associated with baseline-to-EOT changes for MEQ (*η^2^* = 0.222) and MAIA (*η^2^* = 0.192) were rather large.

**Table 4:** Means (standard deviations) of outcome variables at baseline and end of treatment (EOT) for mindfulness-based eating awareness training (MB-EAT) and BEHAVIORAL WEIGHT LOSS (BWL) Groups.

		Baseline	EOT
Outcome variable	Intervention	M (SD)	M (SD)
Body mass index	MB-EAT	33.37 (6.19)	32.91 (6.58)
	BWL	30.71 (5.41)	29.96 (5.46)
Mindful eating questionnaire	MB-EAT	69.00 (11.22)	72.71 (8.56)
	BWL	70.37 (13.73)	78.16 (11.81)
Multidimensional assessment of interoceptive awareness	MB-EAT	88.31 (18.59)	97.53 (23.03)
	BWL	82.86 (24.84)	96.00 (18.64)
Dutch eating behavior questionnaire	MB-EAT	111.43 (17.56)	90.90 (20.53)
	WBL	111.75 (20.13)	101.40 (20.56)
Binge eating scale	MB-EAT	20.00 (7.30)	14.14 (7.60)
	BWL	19.74 (10.52)	13.21 (7.16)
Hospital anxiety and depression scale	MB-EAT	12.14 (5.45)	24.10 (6.37)
	BWL	15.42 (7.31)	27.68 (5.57)

MB-EAT (*n* = 21); BWL (*n* = 20).

**Table 5:** Mixed repeated-measures ANOVA results comparing baseline vs. end of treatment outcomes (EOT) between the mindfulness-based eating awareness training (MB-EAT) and behavioral weight loss (BWL) groups.

	Within (*df* = 1, 39)			Between (*df* = 1, 38)			Interaction (*df* = 1, 39)		
Measure	*F*	*p*	*h^2^*	*F*	*p*	*h^2^*	*F*	*p*	*h^2^*
BMI	7.35	0.010	0.152	2.44	0.126	0.056	0.412	0.525	0.010
MEQ	10.54	0.003	0.210	1.19	0.282	0.030	1.26	0.268	0.032
MAIA	9.28	0.004	0.192	0.38	0.540	0.010	0.29	0.596	0.007
DEBQ	23.44	<0.001	0.375	0.02	0.875	0.001	0.67	0.796	0.002
BES	38.72	<0.001	0.505	0.06	0.805	0.002	0.11	0.739	0.003
HADS	333.42	<0.001	0.898	3.45	0.071	0.083	0.06	0.816	0.001

BMI = body mass index; MEQ = mindful eating questionnaire; MAIA = multidimensional assessment of interoceptive awareness; DEBQ = dutch eating behavior questionnaire; BES = binge eating scale; HADS = hospital anxiety and depression scale.

Also as desired, BMI, binge-eating-related symptoms (BES scores), as well as restrained, emotional, and external eating (DEBQ scores) decreased significantly and substantively from baseline to EOT, with the effect sizes ranging from large to very large: BMI (*η^2^* = 0.152); DEBQ (*η^2^* = 0.375); BES (*η^2^* = 0.505). The most surprising and concerning change from baseline to EOT was found for symptoms of depression and anxiety (HADS scores). That is, participants reported significantly higher symptom severity at EOT than at baseline, and this effect size was the largest in the study (*h^2^* = 0.898).

## 4. Discussion

Given the need created by COVID-19 confinements to recur to telehealth to provide mental and behavioral health services, we designed a RCT that tested the comparative efficacy of two remotely delivered interventions, MB-EAT and BWL. We believe the participant recruitment process was successful as most of those who responded to our call followed through with the screening processes, completed the baseline measures, and enrolled in the study (98 of 135 or about 76%). On the other hand, following initial enrollment, we observed very high rates of attrition in both arms of the study (about 58%). Although these attrition rates are over twice as high as those reported in other comparable, but presential RCT investigations (Miller et al., 2012; Miller et al., 2016), our high attrition rates were also in line with those reported in other relevant RCT investigations physically delivered in person (Kristeller et al., 2014; Salvo et al., 2022). Thus, it is not a given that the high attrition we saw was necessarily or solely due to the remote nature of the intervention.

Our pre-to-post design allowed us to examine whether and how the participants we lost to attrition differed from those who remained in treatment. We learned that the attrition and retained groups were highly similar regarding some demographic and treatment-outcome variables, but those in the attrition group were younger and significantly reported greater levels of binge eating and depression/anxiety symptoms. The negative correlation between age and binge eating, as well as the positive correlation between binge-eating and mental health distress (depression and anxiety), makes it difficult to separate the relative, independent impacts of these three variables in attrition. Given that the only significant predictor of attrition in the logistic regression model was binge-eating symptoms, perhaps this was the most consequential attrition factor.

Unfortunately, there is not much in the way of systematic research in attrition and weight loss interventions to help us interpret our results (Moroshko et al., 2011). Nonetheless, our results coincide with what few other investigators have reported. In their meta-analysis, Moroshko et al. (2011) concluded that young age was the only variable that emerged as a significant but inconsistent predictor of attrition. If we narrow the focus to MB-EAT studies, we found two studies that like us reported that binge-eating symptomatology at baseline was a significant predictor of treatment attrition (Kristeller et al., 2014; Salvo et al., 2022). Regarding depression and anxiety, Salvo et al. (2022) also found that baseline depression and anxiety were higher in those lost to attrition than in those retained. As previous researchers before us, we did not have the foresight to plan for follow-up interviews and focus groups to understand the reasons why some participants remained, and others left. Future studies should conduct follow-up assessments to understand the reasons why different participants might be at a higher attrition risk or what elements of the treatment impeded their continuance.

Except for depression and anxiety symptoms, all pre-to-post, measured changes revealed improvements in eating-related outcomes. That is, participants reported pre-to-post-treatment increases in both eating mindfulness and interoceptive body awareness. Also as hoped for, participants reported reductions in problematic (emotional, external, and restricted) eating, reductions in binge eating and related symptoms, and modest weight (BMI) losses. In line with previous research (Kristeller et al., 2014; Martin et al., 2017), the favorable outcomes we observed were similar across the MB-EAT and the active control intervention. Kristeller et al. (2014) compared the effects of MB-EAT with a cognitive-behavioral psychoeducation control group and a wait-list control. This study found no significant differences between treatments in outcome gains observed in both their MB-EAT and psychoeducational interventions (Kristeller et al., 2014). In a similar study, Martin et al. (2017) compared caloric intake changes across three intervention groups, Mindful Eating, Mindful Decision-Making, and Standard Behavioral. Like us and Kristeller et al. (2014), Martin et al. (2017) did not find significantly different outcomes between any of their interventions. Thus, to our knowledge, we have replicated pre-to-post treatment gains consistently reported in other research, but we are the first to successfully replicate the findings by delivering the interventions remotely via teleconference.

Beyond noting the similarity between our findings and prior research (i.e., Kristeller et al., 2014; Martin et al., 2017; Smith et al., 2018), we can only speculate about the reasons why participants in the BWL intervention experienced similar improvements in outcome variables that were singularly targeted by MB-EAT. One possible explanation is that cognitive-behavioral interventions facilitate internal awareness and mindfulness because participants are taught to identify and modify the factors that control their eating behavior. That is, behavior modification necessarily requires alertness and conscious intention to identify cue-dependent behavior to break entrenched habits and create new ones. We would even argue that for behavior modification to work mindful awareness is a must. Accepting this argument would lead to the conclusion that mindful awareness can be achieved through different means, not solely through explicitly labeled mindful-based practices.

It is important to emphasize that all our pre-to-post-treatment effects were large. Even the smallest among the outcome effects, modest BMI decreases were impressive considering that during the COVID-19 pandemic, BMI increased at the population level across the world (e.g., Di Renzo et al., 2020; Sidor and Rzymski, 2020; Ammar et al., 2021). Placing ourselves in Chile, during the first months of the pandemic (April–May) 25.6% of men and 38.1% of women reported weight gain (Reyes-Olavarría et al., 2020). As widely reported, Chileans gained an average of 8 kg between March and August 2020, ranking a close second in the world among the countries that had gained the most weight (IPSOS, 2021). This increase in weight and BMI was mostly generated by people with a pre-pandemic history of overweight or obesity (Almandoz et al., 2020). Within this context, the modest weight loss of participants who remained in the study becomes very substantive and supports the efficacy of our remotely delivered interventions. We can apply a similar argument to amplify the other positive gains observed in the study, namely the replacement of unhealthy for healthy eating habits and reductions in binge-eating symptoms. For example, a meta-analysis of 26 studies found that eating disorder and obesity-associated symptoms increased by 65% during COVID-19 (Sideli et al., 2021).

The finding that depression and anxiety symptoms increased from baseline to EOT is concerning, particularly because this novel finding is the opposite of what other researchers have reported in related MB-EAT investigations (i.e., Kristeller et al., 2014; Miller et al., 2014; Salvo et al., 2022). We believe these increments in psychological distress were largely reflecting COVID-19 population-based changes. The three intervention cycles took place virtually during the most pronounced spikes in COVID-19 infections observed in Chile. Longitudinal, epidemiological researchers have reported significant increases in anxiety and depression during COVID-19 in Chile (e.g., Duarte and Jiménez-Molina, 2022), and many studies across widely different samples have demonstrated that fear of contracting COVID-19 exacerbated symptoms of anxiety and depression worldwide (e.g., Fitzpatrick et al., 2020; Moore et al., 2021; Rettie and Daniels, 2021). Thus, it is likely that the increments in psychological distress we observed among our participants in part reflected normative distress changes observed during COVID-19.

### 4.1. Strengths, limitations, and conclusions

Our study has a few remarkable strengths, including the use of an RCT design to test the efficacy of a relatively new and promising intervention (MB-EAT), the inclusion of a proven efficacious intervention as the control arm (BWL), and the novelty of delivering both interventions remotely to a sample also recruited remotely. In addition, differential efficacy analyses were likely free of differential history, therapist-characteristics, and participant-characteristics confounding effects because both interventions were conducted temporarily in parallel, by the same team of expert clinicians, and randomization of participants to the two treatment interventions was successful (relevant characteristic were not different between the two groups). Finally, the statistical analyses were conclusive in that there were no “marginal” results and the statistically significant effects were unambiguously large. That is, our investigation is the first to show that remotely administered weight-loss/healthy eating group interventions can be effective, at least at EOT.

On the other hand, our study also has important limitations. Perhaps the most notable is that we do not have long-term follow-up data to examine the extent to which the gains observed at EOT were retained in general and differentially. In addition, we have already noted our relatively high attrition rates, the almost entirely female sample, and the potential iatrogenic effects of the remote interventions on depression and anxiety symptoms. In hindsight, the omission of a waitlisted control group prevented us from testing whether the effects reported could be attributed to generalized COVID-19 effects. Finally, we relied on self-reported height and weight to calculate BMI, which usually results in underestimating measured BMI (e.g., Pursey et al., 2014). On the other hand, underestimations are often the result of minor self-reporting errors, as participants are usually very accurate in reporting their height and weight with correlations between measured and self-reported height, weight, and BMI being consistently above 0.98 (Pursey et al., 2014). Nonetheless, given the novelty of the investigation, we believe these important shortcomings do not invalidate the importance of demonstrating the delivery of MB-EAT and BWL group interventions is potentially efficacious. In addition, the realization of the study limitations revealed important gaps in the literature, such as the need to systematically investigate attrition rates in weight-loss/healthy eating treatments, or the importance of including both, active and inactive treatment controls in RCT studies.

## Data availability statement

The raw data supporting the conclusions of this article will be made available by the authors, without undue reservation.

## Ethics statement

The studies involving human participants were reviewed and approved by Comité Ético de Investigación Universidad Adolfo Ibáñez. The patients/participants provided their written informed consent to participate in this study.

## Author contributions

CU, CC-M, and ÁQ conceptualized and designed of the study. DD-T, MG, and AG directed the execution of the study and including data gathering. CU and AC-B organized the database, performed the statistical analyses, and wrote the first draft of the manuscript. All authors contributed to the article and approved the submitted version.

## Funding

This work was supported by Project: FONDECYT POSTDOCTORADO 2020–2022, N°: 3200764 (Fondo Nacional de Desarrollo Científico y Tecnológico), financed by ANID (Agencia Nacional de Investigación y Desarrollo), Chile.

## Conflict of interest

The authors declare that the research was conducted in the absence of any commercial or financial relationships that could be construed as a potential conflict of interest.

## Publisher’s note

All claims expressed in this article are solely those of the authors and do not necessarily represent those of their affiliated organizations, or those of the publisher, the editors and the reviewers. Any product that may be evaluated in this article, or claim that may be made by its manufacturer, is not guaranteed or endorsed by the publisher.
